# A new improved maximal relevance and minimal redundancy method based on feature subset

**DOI:** 10.1007/s11227-022-04763-2

**Published:** 2022-08-30

**Authors:** Shanshan Xie, Yan Zhang, Danjv Lv, Xu Chen, Jing Lu, Jiang Liu

**Affiliations:** 1 College of Big Data and Intelligent Engineering, Southwest Forestry University, Kunming, 650224 China; 2 College of Mathematics and Physics, Southwest Forestry University, Kunming, 650224 China; 3 Research Institute of Forestry Policy and Information, Chinese Academy of Forestry, Beijing, 100091, China

**Keywords:** mRMR, Feature subset, Feature selection, ImRMR, Sequence forward search

## Abstract

Feature selection plays a very significant role for the success of pattern recognition and data mining. Based on the maximal relevance and minimal redundancy (mRMR) method, combined with feature subset, this paper proposes an improved maximal relevance and minimal redundancy (ImRMR) feature selection method based on feature subset. In ImRMR, the Pearson correlation coefficient and mutual information are first used to measure the relevance of a single feature to the sample category, and a factor is introduced to adjust the weights of the two measurement criteria. And an equal grouping method is exploited to generate candidate feature subsets according to the ranking features. Then, the relevance and redundancy of candidate feature subsets are calculated and the ordered sequence of these feature subsets is gained by incremental search method. Finally, the final optimal feature subset is obtained from these feature subsets by combining the sequence forward search method and the classification learning algorithm. Experiments are conducted on seven datasets. The results show that ImRMR can effectively remove irrelevant and redundant features, which can not only reduce the dimension of sample features and time of model training and prediction, but also improve the classification performance.

## Introduction

With the development of machine learning and artificial intelligence, more and more rich information is obtained from research objects, but some irrelevant and redundant features are added, which leads to higher feature dimensions. For the classification learning, the good learning samples are the key to train the classifier. The irrelevant or redundant information of the samples will increase the complexity of the classification algorithm as well as the time of training and prediction, and directly affect the classification performance. Therefore, it is necessary to select the optimal features that can best represent the characteristic of object, and eliminate irrelevant and redundant features to improve the efficiency of the classifier [1].

Feature selection is the process of selecting the optimal feature subset from the raw feature set to reduce the dimension of the feature space [2]. Its purpose is to simplify the data structure, interpret the data information, and improve the robustness, stability and identification of the model. According to whether the evaluation criteria of feature subset are related to classifier, feature selection methods are divided into three ways: filter, wrapper and embedded [3]. Filter method relies on the properties of the feature space itself, based on feature metrics to maximize data representation information. The feature selection and training classifier are two independent processes. For example, Zhao et al. [4] applied constraint score method to score audio features in environmental sound classification and select the optimal feature subset to improve the classification performance. Saqlain et al. [5] used fisher score method to select feature subsets, and transferred selected feature subsets to the RBF kernel-based SVM for diagnosing heart disease. Yong et al. [6] divided features into three levels: strong correlation, sub-strong correlation and other features, based on the measurement criterion of mutual information and the correlation between the feature and the label. Then the features are simplified. Wrapper method relies on preselected classifiers, takes the performance of the classifier as an evaluation criterion for feature subset, and removes features that negatively affect the classification performance. For example, Ye et al. [7] proposed a multimodal wrapper feature selection method based on effective distance, which considered the global relationship between samples. Mafarja et al. [8] wrapped the whale optimization algorithm into the classifier, and used the crossover and mutation characteristics of the optimization algorithm to enhance the speed and accuracy of feature search. In order to improve the classification performance of the financial credit scoring model, Jadhav et al. [9] proposed an information gain directed feature selection algorithm, and propagated the top *m* features through the genetic algorithm wrapper. The embedded method puts the feature selection process into the learning algorithm, which is a compromise between the filter method and the wrapper method. For example, Xu et al. [10] proposed a joint intra-class variation coefficient and inter-class mutual information, constructed a joint index to evaluate the feature contribution, and combined the embedded method for feature selection. Maldonado et al. penalized the cardinality of feature sets through the scale factor technology, and used SVM as the embedded base classifier for feature selection. This method was applied to high-dimensional datasets with extremely uneven categories [11]. Liu et al. [12] proposed an unsupervised adaptive neighborhood embedded feature selection method, which mainly captures the internal geometric structure of the data based on the *K*-nearest neighbor method. Because both the wrapper and embedded methods have certain dependencies on the classification learning algorithm, and are prone to problems such as overfitting and low efficiency. The filter method has high efficiency and is suitable for various data types. The method has strong versatility and low algorithm complexity.

In the filter method, the feature metric is the key factor. That the maximal relevance and minimal redundancy (mRMR) criterion is a filter feature metric for pattern recognition [13], it aims to select the feature subset with the maximal dependency, maximal relevance and the minimal redundancy from the raw feature set. However, mRMR only considered the contribution of a single feature. The feature with the maximum contribution from the raw feature set are selected and added to form the optimal feature subset. In this way, the relevance between features will be ignored, and the joint contribution of multiple features to the classification will not be considered enough, resulting in the selected feature subset may not be optimal. There is still a certain room for improvement in the selection of the optimal feature subset. Therefore, based on the mRMR method, in this paper, the ImRMR (Improved mRMR) method is proposed. First, the relevance between each feature and the class is calculated through the Pearson correlation coefficient and mutual information, and tradeoff the two metrics to rank the features. Secondly, candidate feature subsets are generated with the proposed equal grouping method (EGM) according to the ranking features. The joint contribution of feature subset is evaluated by the relevance and redundancy of feature subset, and the ranking of feature subsets is found by incremental search method. Finally, the final optimal feature subset is obtained by combining the sequence forward search method (SFS). The experiments are conducted on seven datasets to verify the effectiveness of the ImRMR.

This paper adopts a supervised learning method to perform feature selection on these datasets through ImRMR, and uses advanced machine learning classification algorithm (random forest) to classify these features. In this study, 70% of each dataset was randomly selected for the training dataset, and the remaining 30% was used for the test dataset. We conduct extensive experiments and evaluate on various performance metrics (accuracy, dimensionality reduction rate, comprehensive rate, precision, recall and *F*-measure) to determine the effectiveness of the proposed method. Experiments show that the proposed feature selection method on random forest classifier outperforms the original mRMR and other methods.

The rest of this paper is organized as follows: Sect. 2 lists the related work done by various authors. Section 3 describes the mRMR method. Section 4 presents the proposed ImRMR method in detail. Then, the experiments and results are analyzed in Sect. 5. Finally, we give limitations and conclusion.

## Related work

Based on the mRMR, Lu et al. extracted the key information most relevant to the fault location in traditional transmission line faults. By mining the implicit relationship between key features and fault location, the fault location result is obtained by synthesizing multiple feature information. This method has better fault location accuracy and better adaptability to transient components that appear after the fault [14]. Billah and Waheed [15] effectively reduced the dimensionality of the extracted features of endoscopic video frames with mRMR. Toaar et al. used the mRMR to select the features of the lung X-ray images for the diagnosis of pneumonia extracted by three deep models, and combined them to form an efficient feature set. The selected feature set provided robust and consistent features for pneumonia detection, and the mRMR can effectively reduce the dimension of the feature set [16]. Gu et al. calculated the relevance between power transformer fault feature quantities and the redundancy between feature quantities and fault types based on the mRMR, the optimal transformer fault feature set was obtained. Experiments show that the optimal feature set is more efficient than the traditional feature set in transformer fault diagnosis [17]. Erolu et al. [18] used the mRMR to select the features of breast ultrasound image extracted by the hybrid CNN structure, and achieved good results in classification and recognition. Fan et al. [19] used mRMR to select important lead-rhythm features extracted from electroencephalogram recordings to build a predictive model to predict the prognostic effect of acupuncture on depression. Tuncer et al. [20] used the discrete wavelet transform to decompose the electroencephalogram signal, and then used the mRMR to select the most discriminant feature from the texture features generated by the decomposed discrete wavelet transform subband, and used SVM classifier to classify the selected features. In the study of COVID-19 disease classification using supervised optimization machine learning technology, Sharma et al. [21] used the mRMR to remove irrelevant and misleading features in the high-dimensional data of COVID-19 to reduce the size of the search space of the cuckoo search algorithm and improve the learning efficiency. Baliarsingh et al. employed mRMR to select relevant subsets of genes from the microarray dataset. Then, simulated annealing is hybridized with Rao algorithm to improve the solution quality after each iteration of Rao algorithm. The discriminant genes selected on the SRBCT dataset have high classification accuracy [22].

In addition, many scholars have improved the mRMR to obtain better feature selection results. Feng and Zhang improved the mRMR by using conditional mutual information formula and three-dimensional calculation to determine the candidate connection of each attribute node in Bayesian network classifier. The method enhanced the reliability and robustness of small sample calculation [23]. Yao et al. introduced mRMR into the particle swarm optimization algorithm search process to select feature subset. The feasibility and effectiveness of the proposed algorithm were verified on the UCI dataset with SVM as the classifier [24]. Li and Wang [25] proposed a new mRMR method to use a variety of different evaluation criteria to measure the redundancy between features and the relevance between features and categories, and an indicator vector *λ* was introduced to describe the actual data dimension requirements of users. Wang et al. [26] improved the mRMR by merging relevance measurement coefficients to obtain a primary feature subset, and binary-coded the feature subset, and then combined the genetic algorithm to search for the optimal or suboptimal feature subset. Jo et al. [27] used the Pearson correlation coefficient as the redundancy metric and the *R* value as the relevance metric to improve the mRMR. Ahmed et al. put forward an enhanced mRMR (EmRMR) filtering method to remove the noise features in ransomware, and selected the most relevant feature subset to characterize the real behavior of ransomware. EmRMR requires only a small amount of evaluation, avoiding unnecessary calculations inherent in the original mRMR [28]. Combines EmRMR with term frequency-inverse document frequency (TF-IDF), a weighted mRMR (WmRMR) technology was proposed to filter out runtime noise behavior according to the weight calculated by TF-IDF. Compared with the mRMR, WmRMR has low-dimensional complexity and less evaluation times, and better estimates the feature significance in the data captured in the early stage of ransomware attacks [29].

The existing improved methods for mRMR are based on the calculation of correlation and redundancy of a single feature, they ignore the joint contribution of multiple features, and do not take into account the calculation of redundancy and correlation between feature subsets. We propose an improve mRMR (ImRMR) based on feature subsets. The proposed method and performance evaluation will be discussed in the following sections.

## mRMR feature selection

### mRMR definition

Maximal relevance and minimal redundancy (mRMR) is a filter feature measurement criterion, which calculates the redundancy between features and the correlation between features and class based on mutual information $I\left( {x;y} \right)$. It selects the features that are most relevant to the category and have the least redundancy with other features from the raw feature set.

The mutual information $I\left( {x;y } \right)$ is defined as Eq. 1.

1 $$I\left( {x;y} \right) = \iint {p\left( {x,y} \right)\log \frac{{p\left( {x,y} \right)}}{p\left( x \right)p\left( y \right)}{\text{d}}x{\text{d}}y}$$

where $p\left( {x,y} \right)$ is the joint probability density of random variables $x$ and $y$, and $p\left( x \right)$ and $p\left( y \right)$ are the marginal probability densities of $x$ and $y$, respectively.

Given a sample feature set $S = \{ f_{1} ,f_{2} , \ldots f_{n} \}$ and a sample class $c$. The relevance between $S$ and $c$ is the mean of all mutual information between each feature $f_{i}$ and class $c$. It is shown in Eq. 2.

2 $$D\left( {S,c} \right) = \frac{1}{\left| s \right|}\mathop \sum \limits_{{f_{i} \in S}} I\left( {f_{i} ;c} \right)$$

where $\left| S \right|$ is the number of features in $S$, and $I\left( {f_{i} ;c} \right)$ is the mutual information between the feature $f_{i}$ and the class $c$.

The redundancy of all features in $S$ is the mean of all mutual information between feature $f_{i}$ and feature $f_{j}$. It is shown in Eq. 3.

3 $$R\left( S \right) = \frac{1}{{\left| s \right|^{2} }}\mathop \sum \limits_{{f_{i} ,f_{j} \in S}} I\left( {f_{i} ;f_{j} } \right)$$

The mRMR method seeks the optimal features of the samples with maximal relevance $D\left( {S,c} \right)$ and minimal redundancy $R\left( S \right)$. So the criterion of feature measurement for mRMR can be as shown in Eq. 4.

4 $${\text{mRMR}} = \mathop {\max }\limits_{s} \left( {D\left( {S,c} \right) - R\left( S \right)} \right)$$

### Incremental search method

Incremental search is used to quickly and efficiently select the optimal feature set. Given the raw feature set $X$, if the optimal feature $S_{m - 1}$ has been selected, then it will continue to search for the optimal feature in the remaining feature space $X - S_{m - 1}$. Equation 4 can be illustrated as Eq. 5.

5 $$\mathop {\max }\limits_{{f_{j} \in X - S_{m - 1} }} \left[ {I\left( {f_{i} ;\,c} \right) - \frac{1}{m - 1}\mathop \sum \limits_{{f_{i} \in S_{m - 1} }} I\left( {f_{j} ;f_{i} } \right)} \right]$$

## Improved mRMR based on feature subset

The mRMR method measures the contribution of feature by calculating the relevance and redundancy of individual feature. The joint contribution of multiple features is ignored. Relevance and redundancy are only based on mutual information measures. Therefore, to obtain the optimal feature subset, from the perspective of feature subset, this paper uses two measurement criteria, Pearson correlation coefficient and mutual information to evaluate the relevance and redundancy of the feature subsets. A weight factor is introduced to tradeoff the two measurement criteria.

**Fig. 1:**
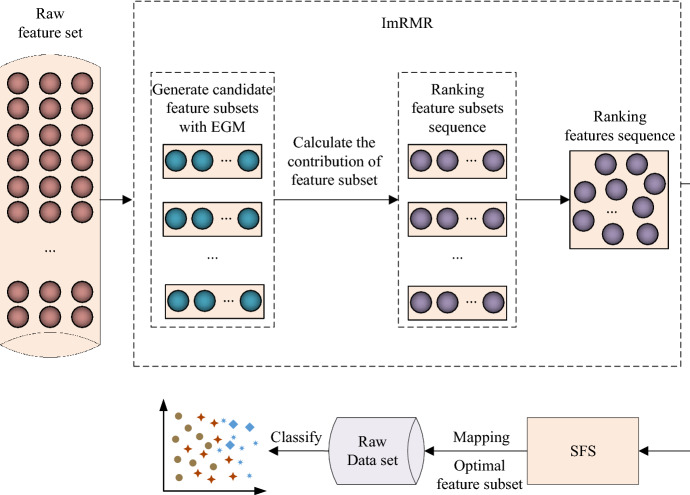
The follow chart of feature selection with ImRMR method

The whole process of proposed ImRMR is shown in Fig. 1. Firstly, we adopt equal grouping method (EGM) to initially divide the candidate feature subsets. Then the contribution of each candidate feature subset is calculated with the correlation and redundancy according to the Pearson correlation coefficient and mutual information. Finally, we convert the ranking of the feature subsets into the feature ranking and combine the SFS search strategy to obtain the final preferred subset. Mapping the optimal feature subset to the raw sample set can carry out classification and recognition.

### Initialize the candidate feature subsets with EGM

Given a sample set of $N*M$, $N$ represents the number of samples, and $M$ refers to the feature dimension. The base decision tree in the random forest includes the feature number $\log_{2} \left( M \right)$ as the candidate feature subsets [30]. The relevance between each feature and the class is calculated by the Pearson correlation coefficient and mutual information, and *α* is introduced to adjust the weight of the two indicators. The features are ranked on relevance. The relevance $Ic\left( {f_{i} ;c} \right)$ between a single feature and the sample class is calculated as shown in Eqs. 6 and 7.

6 $$Ic\left( {f_{i} ;\,c} \right) = \alpha *I\left( {f_{i} ;\,c} \right) + \left( {1 - \alpha } \right)*{\text{corr}}\left( {f_{i} ;\,c} \right)$$

7 $${\text{corr}}\left( {f_{i} ;\,c} \right) = \frac{{\sum f_{i} c - \frac{{\sum f_{i} \sum c}}{N}}}{{\sqrt {\left( {\sum f_{i}^{2} - \frac{{\left( {\sum f_{i} } \right)^{2} }}{N}} \right)\left( {\sum c^{2} - \frac{{\left( {\sum c} \right)^{2} }}{N}} \right)} }}$$

where $\alpha \in \left[ {0.1,1} \right]$, with a step size of 0.1, $I\left( {f_{i} ;\,c} \right)$ represents the mutual information between each feature and class, and ${\text{corr}}\left( {f_{i} ;\,c} \right)$ refers to the Pearson correlation coefficient between each feature and class.

The range of feature subsets $r$ is shown in Eqs. 8 and 9.

8 $$r = \left[ {1,{\text{round}}\left( \frac{M}{Fc} \right)} \right]$$

9 $$Fc = {\text{round}}\left( {\log_{2} \left( M \right)} \right)$$

where $Fc$ is the number of features in the feature subset.

This paper exploits an equal grouping method (EGM) to generate candidate feature subsets. According to the ranking features with relevance, each feature is assigned to a group one by one. When the first-round assignation has finished, the feature will be assigned to a group reversely till the end of the round, and keep going the assignation and swapping directions until all the features assigned to the groups.

**Fig. 2:**
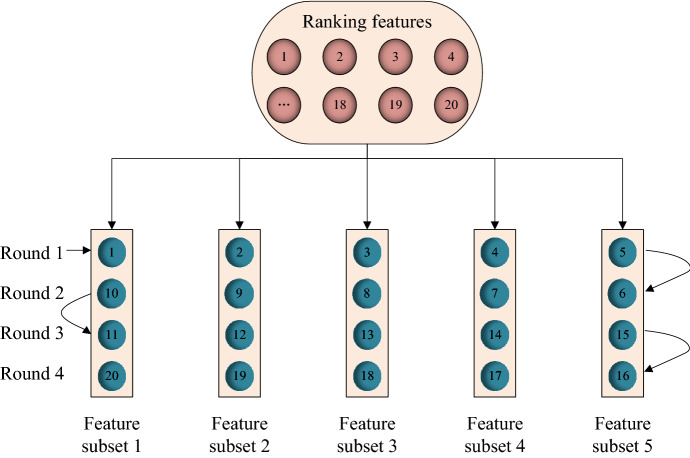
Generate feature subsets with equal grouping method

For example, suppose the number of features $M$ = 20, then $Fc$ = 4, and the range of feature subsets $r$ = 5, the feature subsets generation process is shown in Fig. 2.

### Generate ranking feature subsets sequence

By calculating the contribution of each candidate feature subset, the ranking sequence of these feature subsets is obtained. It is mainly divided into the following three steps.

1. Calculate the relevance of all individual features in each candidate feature subset according to Eq. 6, and accumulate them to obtain the contribution of the candidate feature subset. The candidate feature subset with the largest contribution is taken as the first selected feature subset $F_{y}$;
2. Calculate the relevance $D\left( {F_{x} ;\,c} \right)$ and redundancy $R\left( {F_{x} ;\,F_{y} } \right)$ of the remaining candidate feature subsets according to Eqs. 10 and 12, respectively;
3. Combined with Eq. 14, the incremental search method is used to select feature subset. Then, contribution ranking of all feature subsets is obtained.
  10 $$D\left( {F_{x} ;\,c} \right) = \beta *I\left( {F_{x} ;\,c} \right) + \left( {1 - \beta } \right)*{\text{corr}}\left( {F_{x} ;\,c} \right)$$
  11 $$I\left( {F_{x} ;\,c} \right) = \mathop \sum \limits_{i = 1}^{Fc} I\left( {f_{i} ;\,c} \right),{\text{corr}}\left( {F_{x} ;\,c} \right) = \mathop \sum \limits_{i = 1}^{Fc} {\text{corr}}\left( {f_{i} ;\,c} \right)$$
  12 $$R\left( {F_{x} ;\,F_{y} } \right) = \frac{1}{m - 1}\mathop \sum \limits_{{F_{y} \in S_{m - 1} }} \left( {\beta *I\left( {F_{x} ;\,F_{y} } \right) + \left( {1 - \beta } \right)*{\text{corr}}\left( {F_{x} ;\,F_{y} } \right)} \right)$$
  13 $$I\left( {F_{x} ;\,F_{y} } \right) = \mathop \sum \limits_{i,j = 1}^{Fc} I\left( {f_{i} ;\,f_{j} } \right), {\text{corr}}\left( {F_{x} ;\,F_{y} } \right) = \mathop \sum \limits_{i,j = 1}^{Fc} {\text{corr}}\left( {f_{i} ;\,f_{j} } \right)$$
  14 $${\text{ImRMR}} = \mathop {\max }\limits_{{F_{x} \in X - S_{m - 1} }} \left[ {D\left( {F_{x} ;\,c} \right) - R\left( {F_{x} ;\,F_{y} } \right)} \right]$$
  where $\beta \in \left[ {0.1,1} \right]$, with a step size of 0.1.

### Acquisition of optimal feature subset

Take out the features of the feature subsets sequence in turn to find features sequence. The optimal feature subset is obtained according to the ranking features sequence and the SFS search strategy.

The feature selection algorithm based on ImRMR and SFS is descripted as follows.
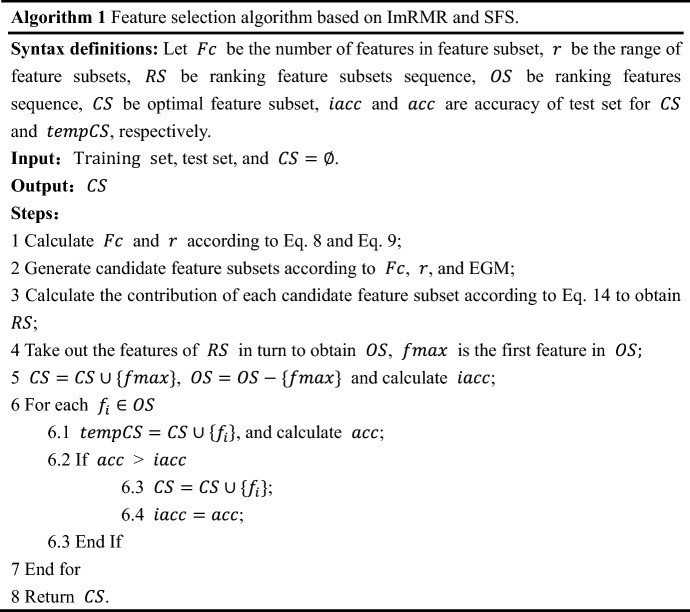


In addition, for the ImRMR algorithm, the same $\alpha$ and different $\beta$ settings make the sample set generate different feature subset when calculating the contribution of feature subset according to Eq. 14. Therefore, the different feature subsets $b_{1} ,b_{2} , \ldots ,b_{m}$ can be obtained, and $m$ represents the number of settings $\beta$, where the feature subset $b_{\max }$ with the highest accuracy is selected as the optimal feature subset for the current $\alpha$.

Different $\alpha$ settings make the sample set generate different optimal feature subset such as $CS_{1} ,CS_{2} , \ldots ,CS_{n}$, $n$ represents the number of settings $\alpha$. The feature subset $CS^{*}$ with the highest accuracy among these $n$ feature subsets is selected as the final optimal feature subset.

## Experiment and result analysis

### Experimental environment

The hardware platform is a laptop computer with 16G RAM and 11th Gen Intel(R) Core(TM) i7-11370H @ 3.30 GHz CPU. The operating system is Windows 10 64-bit. MATLAB 2018b is the data processing, programming and running platform as well WEKA 3.8.5.

### Experimental datasets

Seven datasets were involved in experiments. Five of them are provided by the UCI Machine Learning Repository [31]: Musk dataset (Musk), Urban land cover dataset (Urban), Glass dataset (Glass), Libras Movement dataset (Movement) and Ionosphere dataset (Ionosphere). One dataset is the commonly used hyperspectral dataset Pavia University (PU) [32]. And the other one is crane songs dataset (Crane).

Each dataset is divided into training set and test set as the ratio of 7:3. The information of datasets are listed in Table 1.

**Table 1:** The information of datasets

Dataset	Training set	Test set	Number of features	Number of categories
Musk	334	142	166	2
Urban	478	197	147	9
Ionosphere	246	105	34	2
Glass	150	64	9	6
Movement	252	108	90	15
PU	1800	900	103	9
Crane	243	100	75	7

### The design of experiments

Two groups experimental scheme are designed. One group compared the proposed method of EGM with the method of randomly selecting features to generate candidate feature subsets. And the other compared the ImRMR with other feature selection methods, such as mRMR, InfoGain (IG) [33], Symmetrical Uncert (SU) [34], GainRatio (GR) [35] and ReliefF (RfF) [36].

The four feature selection methods of IG, SU, GR RfF and the classification method of random forest involved in the experiment are all built-in methods in WEKA 3.8.5, which are implemented by MATLAB 2018b calling the interface of WEKA 3.8.5, and the parameters of each feature selection method are default parameters. The mRMR method has no parameter settings, and $\alpha \;{\text{and}}\;\beta$ in ImRMR are set in $\left[ {0.1,1} \right]$.

Random forest is an ensemble learning method based on bagging, which can handle classification problems and regression problems well, and is one of the most widely used machine learning methods at present. To verify effectiveness of the proposed ImRMR feature selection method, the random forest classifier is adopted to obtain classification results of the selected optimal feature subset. Each experiment is repeated 30 times independently, and the average of the experimental results is taken as the final result. The evaluation indicators include accuracy rate, dimensionality reduction rate, comprehensive rate, precision, recall and *F*-measure.

The accuracy rate is used to evaluate the proportion of correctly identified samples to the total number of samples in the prediction results. Its calculation is shown in Eq. 15.

15 $${\text{Acc}} = \frac{{P_{a} }}{N}*100\%$$

where $P_{a}$ is the number of the correctly classified samples, and $N$ represents the number of all samples.

The dimensionality reduction rate as an evaluation indictor is introduced, as shown in Eq. 16.

16 $$Dr = \left( {1 - \frac{Sc}{{Oc}}} \right)*100\%$$

where $Sc$ is the number of selected features, and $Oc$ represents the number of raw features. The larger the $Dr$ value, the stronger the ability to reduce dimensions.

The comprehensive rate considers the accuracy rate and the dimensionality reduction rate, as shown in Eq. 17.

17 $$Z = \theta *{\text{Acc}} + \left( {1 - \theta } \right)*Dr$$

where $Z$ is the comprehensive rate, ${\text{Acc}}$ is the accuracy rate, $Dr$ is the dimensionality reduction rate. The $\theta$ is the tradeoff factor. In experiments, the value of $\theta$ is 0.5.

The precision is used to evaluate the proportion of all predicted correct samples that contain actual correct samples. Its calculation is shown in Eq. 18.

18 $${\text{Precision}} = \frac{{{\text{TP}}}}{{{\text{TP}} + {\text{FP}}}}$$

where TP is the sum of the number of correctly classified samples and FP is the number of samples predicted to be correct that are actually wrong.

The recall rate is the percentage of in all samples where the correct sample is predicted to be correct. Its calculation is shown in Eq. 19.

19 $${\text{Recall}} = \frac{{{\text{TP}}}}{{{\text{TP}} + {\text{FN}}}}$$

where TP is the sum of the number of correctly classified samples and FN is the number of samples that are actually correct but predicted to be wrong.

*F*-measure is precision and recall weighted harmonic mean. *F*-measure calculation is shown in Eq. 20.

20 $$F{\text{-measure}} = 2 \times \frac{{{\text{Precision}} \times {\text{Recall}}}}{{{\text{Precision}} + {\text{Recall}}}}$$

### Result analysis

#### Comparison of EGM with random grouping

In this group experiments, EGM and the method of random grouping are, respectively, applied to the generating candidate feature subsets part of ImRMR, namely ImRMR-EGM and ImRMR-RS. The random grouping method is different from EGM only in that the features of its grouping are randomly assigned, and other parts are consistent with EGM.

**Fig. 3:**
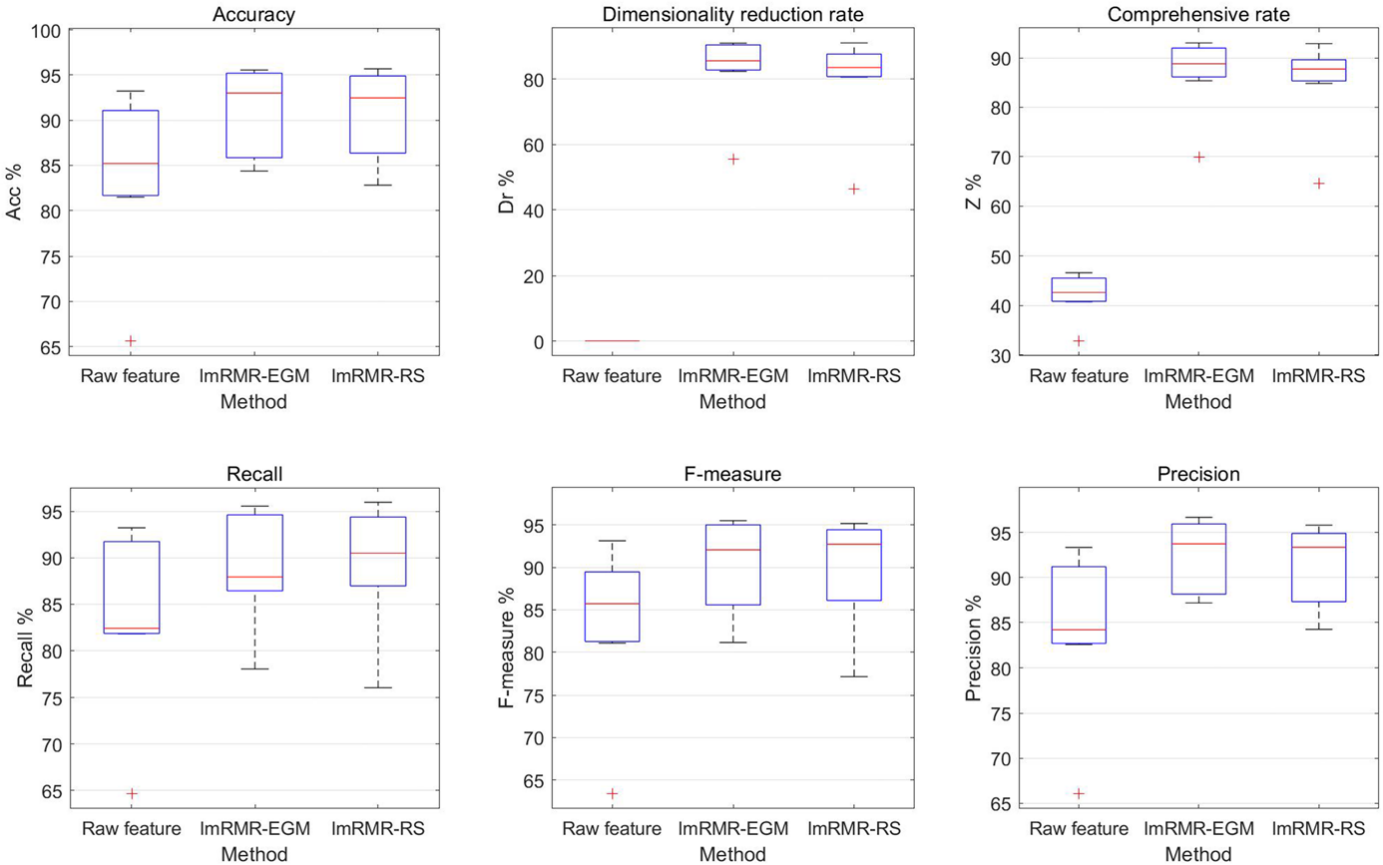
Performance of ImRMR-EGM and ImRMR-RS

Using SFS search strategy, by recording the dimensionality reduction rate, the accuracy rate, precision, recall, *F*-measure and the comprehensive rate when the classification accuracy obtained by the ImRMR-EGM and ImRMR-RS feature selection of the seven datasets reached the maximum value, the performances of the two methods are compared. The experimental results are shown in Table 2 and Fig. 3.

**Table 2:** Experimental results of ImRMR-EGM and ImRMR-RS

Dataset	Performance (%)	Feature selection methods		
		Raw feature	ImRMR-EGM	ImRMR-RS
Musk	Acc	85.21	**95.07**	94.72
	Dr	0.00	90.96	**91.04**
	Z	42.61	**93.02**	92.88
	Recall	92.21	94.81	**95.97**
	*F*-measure	87.12	**95.42**	95.17
	Precision	82.56	**96.05**	94.60
Urban	Acc	82.23	**87.82**	87.02
	Dr	0.00	**90.48**	88.41
	Z	41.12	**89.15**	87.72
	Recall	82.40	**87.93**	87.43
	*F*-measure	81.81	**88.03**	87.02
	Precision	84.18	**89.35**	87.99
Ionosphere	Acc	91.43	95.24	**95.68**
	Dr	0.00	**82.35**	80.59
	Z	45.71	**88.80**	88.14
	Recall	81.82	87.88	**90.50**
	*F*-measure	85.71	92.06	**93.05**
	Precision	90.00	**96.67**	95.80
Glass	Acc	65.63	**84.38**	82.81
	Dr	0.00	**55.56**	46.30
	Z	32.81	**69.97**	64.55
	Recall	64.62	**78.08**	76.06
	*F*-measure	63.36	**81.15**	77.18
	Precision	66.02	**87.74**	84.24
Movement	Acc	81.48	85.19	**86.14**
	Dr	0.00	**85.56**	83.52
	Z	40.74	**85.37**	84.83
	Recall	81.94	85.96	**86.81**
	*F*-measure	81.08	84.75	**85.79**
	Precision	83.03	**87.17**	87.08
PU	Acc	93.22	**95.56**	94.94
	Dr	0.00	**90.29**	85.28
	Z	46.61	**92.92**	90.11
	Recall	93.22	**95.56**	94.94
	*F*-measure	93.13	**95.50**	94.89
	Precision	93.33	**95.56**	94.98
Crane	Acc	90.00	**93.00**	92.47
	Dr	0.00	**84.00**	81.16
	Z	45.00	**88.50**	86.81
	Recall	90.32	**94.02**	92.70
	*F*-measure	90.24	**93.73**	92.72
	Precision	91.59	**93.72**	93.35

Best performance in bold

Seen from Table 2 and Fig. 3, ImRMR-EGM has good effects on the seven datasets. The dimensionality reduction rate reaches 55.56–90.96%, and the classification accuracy is 2.34–18.75% higher than that of the raw feature. ImRMR-EGM not only effectively reduces the dimension of the dataset and but also improves the classification accuracy.

In seven datasets, the comprehensive rate of ImRMR-EGM is higher than that of ImRMR-RS by 0.14–5.42%. And in most cases, the accuracy, precision, recall and *F*-measure of ImRMR-EGM are higher than ImRMR-RS. Therefore, the EGM has achieved good results in generating candidate feature subsets, which outperforms the random grouping method.

#### Comparison of ImRMR with other feature selection methods

Experiments with ImRMR and other five feature selection methods are conducted on seven datasets. Combine SFS search strategy, the results of various feature selection methods are classified by random forest. Their performances are compared with the evaluation indices including dimensionality reduction rate, accuracy rate, precision, recall, *F*-measure and comprehensive rate when the classification accuracy reaches the maximum.

**Fig. 4:**
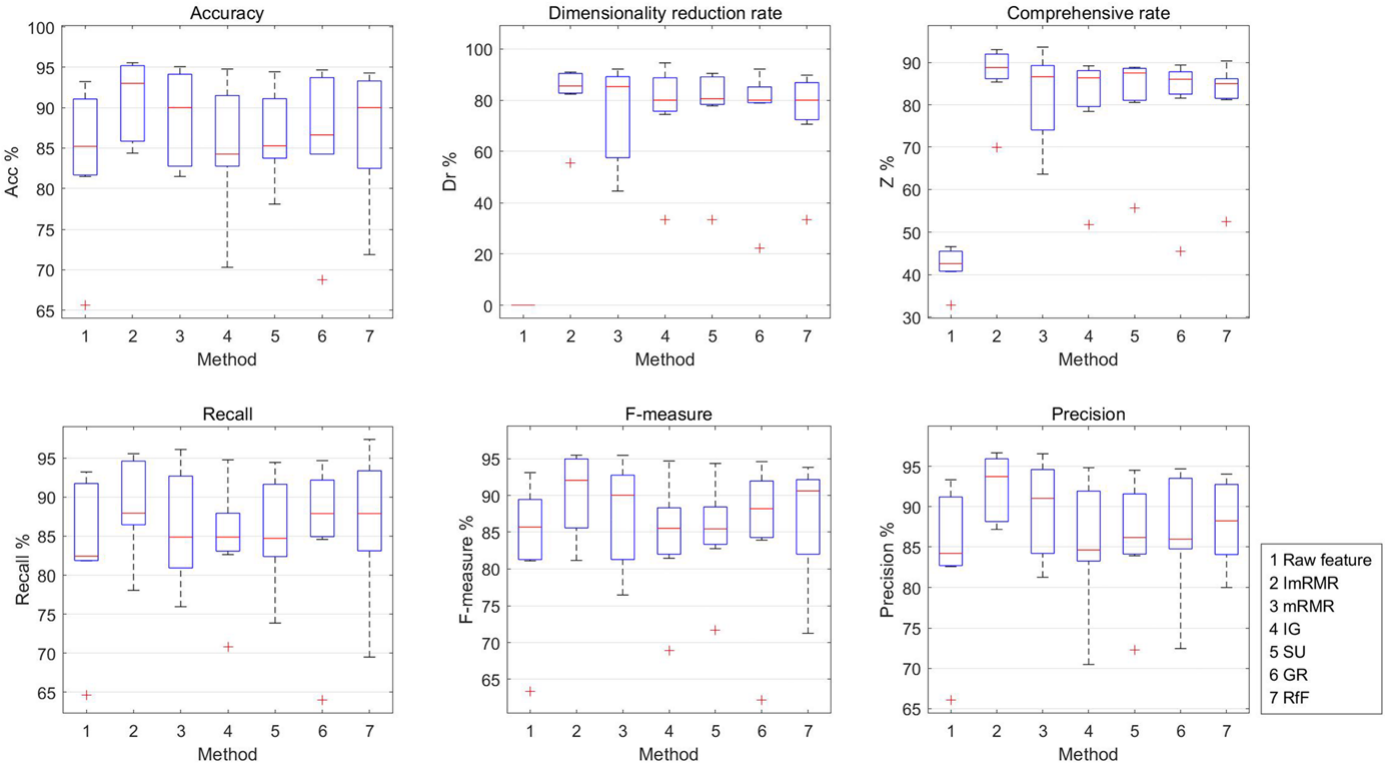
Performance of ImRMR with other methods

The experimental results are shown in Table 3 and Fig. 4. And the abscissa labels 1, 2, 3, 4, 5, 6, and 7 in Fig. 4 represent raw feature and six feature selection method of ImRMR, mRMR, IG, SU, GR, and RfF, respectively.

**Table 3:** Experimental results of ImRMR and other five methods

Dataset	Performance (%)	Raw feature	Feature selection methods					
			ImRMR	mRMR	IG	SU	GR	RfF
Musk	Acc	85.21	**95.07**	**95.07**	83.80	87.32	86.62	91.55
	Dr	0.00	90.96	92.17	**94.58**	90.36	92.17	89.16
	Z	42.61	93.02	**93.62**	89.19	88.84	89.39	90.35
	Recall	92.21	94.81	96.10	88.31	93.51	92.21	**97.40**
	*F*-measure	87.12	95.42	**95.48**	85.53	88.89	88.20	92.59
	Precision	82.56	**96.05**	94.87	82.93	84.71	84.52	88.24
Urban	Acc	82.23	**87.82**	82.74	84.26	85.28	84.26	82.74
	Dr	0.00	90.48	90.48	**91.16**	90.47	86.39	89.80
	Z	41.12	**89.15**	86.61	87.71	87.88	85.33	86.27
	Recall	82.40	**87.93**	80.62	84.37	84.70	85.98	83.08
	*F*-measure	81.81	**88.03**	82.08	83.64	85.14	85.38	82.66
	Precision	84.18	**89.35**	86.48	84.61	86.99	85.96	84.22
Ionosphere	Acc	91.43	**95.24**	94.29	93.33	92.38	94.29	94.29
	Dr	0.00	82.35	**85.29**	79.41	**85.29**	79.41	70.59
	Z	45.71	88.80	**89.79**	86.37	88.84	86.85	82.44
	Recall	81.82	**87.88**	84.85	84.85	81.82	**87.88**	**87.88**
	*F*-measure	85.71	**92.06**	90.32	88.89	87.10	90.63	90.63
	Precision	90.00	**96.67**	96.55	93.33	93.10	93.55	93.55
Glass	Acc	65.63	**84.38**	82.81	70.31	78.13	68.75	71.88
	Dr	0.00	**55.56**	44.44	33.33	33.33	22.22	33.33
	Z	32.81	**69.97**	63.63	51.82	55.73	45.49	52.60
	Recall	64.62	**78.08**	75.99	70.80	73.88	63.96	69.51
	*F*-measure	63.36	**81.15**	76.50	68.95	71.71	62.13	71.28
	Precision	66.02	**87.74**	81.23	70.47	72.28	72.44	80.02
Movement	Acc	81.48	**85.19**	81.48	82.41	83.33	84.26	82.41
	Dr	0.00	**85.56**	75.56	74.44	77.78	78.89	80.00
	Z	40.74	**85.37**	78.52	78.43	80.56	81.57	81.20
	Recall	81.94	**85.96**	81.71	82.61	83.96	84.56	83.10
	*F*-measure	81.08	**84.75**	81.00	81.44	82.75	83.91	81.78
	Precision	83.03	**87.17**	83.42	84.17	83.90	85.45	84.00
PU	Acc	93.22	**95.56**	93.67	94.78	94.44	94.67	93.89
	Dr	0.00	**90.29**	51.46	81.55	80.58	81.55	77.67
	Z	46.61	**92.92**	72.56	88.17	87.51	88.11	85.78
	Recall	93.22	**95.56**	93.67	94.78	94.44	94.67	93.89
	*F*-measure	93.13	**95.50**	93.59	94.71	94.37	94.62	93.84
	Precision	93.33	**95.56**	93.76	94.83	94.51	94.68	94.03
Crane	Acc	90.00	**93.00**	90.00	86.00	85.00	92.00	90.00
	Dr	0.00	84.00	**85.33**	80.00	80.00	80.00	80.00
	Z	45.00	**88.50**	87.67	83.00	82.50	86.00	85.00
	Recall	90.32	**94.02**	89.73	86.72	86.00	92.06	91.80
	*F*-measure	90.24	**93.73**	90.03	86.63	85.44	92.43	90.90
	Precision	91.59	**93.72**	91.03	87.70	86.17	93.38	90.36

Best performance in bold

Seen from Table 3 and Fig. 4, ImRMR outperforms five methods (mRMR, IG, SU, GR, and RfF) on seven datasets of Musk, Urban, Ionosphere, Glass, PU, Movement, and Crane. Among them, the comprehensive rate of ImRMR is 1.27–3.82%, 6.34–24.48%, 3.8–6.94%, 4.75–20.36% and 0.83–6% higher than the five methods on the five datasets of Urban, Glass, Movement, PU and Crane, respectively; for the Musk dataset, the comprehensive rate of ImRMR is only 0.6% lower than that of mRMR, and 2.67–4.18% higher than that of the other four methods; for the Ionosphere dataset, the comprehensive rate of ImRMR is only 0.99% and 0.04% lower than mRMR and SU, respectively, and 1.95–6.36% higher than the other three methods. The accuracy of ImRMR on six datasets (Urban, Ionosphere, Glass, PU, Movement and Crane) is higher than these five feature selection methods, and the accuracy on Musk datasets is the same as mRMR and better than the other four feature selection methods. In addition, in most cases, ImRMR is superior to the other five feature selection methods in terms of recall, *F*-measure and precision on seven sets of datasets. Therefore, from a comprehensive comparison, the ImRMR feature selection method is superior to the five feature selection methods.

From the above comparative experiments, experimental results show that the proposed ImRMR method can effectively remove irrelevant and redundant features, which can not only reduce the dimension of sample features, but also achieve better classification and recognition results.

#### Comparison with state-of-the-art methods

Recently, various researchers have analyzed various feature selection methods due to improved accuracy results. Table 4 shows the comparative analysis of the proposed method with other methods using the same dataset. It can be noticed that the proposed method shows higher accuracy (Acc) and comprehensive rate (Z) in feature selection compared to other methods using the same dataset.

**Table 4:** Comparative analysis with other methods

Study	Year	Method	Dataset	Acc (%)	Dr (%)	Z (%)
Mafarja et al. [8]	2018	Whale optimization approaches for wrapper feature selection with KNN classifier	Ionosphere	92.56	57.60	75.08
Mafarja et al. [37]	2019	Binary grasshopper optimization algorithm with mutation with KNN classifier	Ionosphere	**96.59**	79.41	88
Du et al. [38]	2020	Improved binary symbiotic organism search algorithm with transfer functions with KNN classifier	Ionosphere	92.96	–	–
Xu et al. [10]	2020	Maximum feature tree embedded with mutual information and coefficient of variation with random forest classifier	Ionosphere	94.32	64.71	79.52
Ghosh et al. [39]	2020	Binary social mimic optimization algorithm with x-shaped transfer function with KNN classifier	Ionosphere	95.71	76.47	86.09
Han et al. [40]	2021	Multi-objective particle swarm optimization with adaptive strategies with KNN classifier	Ionosphere	89.18	–	–
Kang et al. [41]	2022	Grey wolf improved flower pollination algorithm with KNN classifier	Ionosphere	95.36	76.18	85.77
Proposed method	2022	ImRMR with random forest classifier	Ionosphere	95.24	**82.35**	**88.80**
Xu et al. [10]	2020	Mutual information and coefficient of variation with random forest classifier	Crane	91.00	45.33	68.17
Proposed method	2022	ImRMR with random forest classifier	Crane	**93.00**	**84.00**	**88.50**
Zhang et al. [42]	2018	Maximum joint mutual information algorithm with KNN classifier	Movement	80.67	3.33	42.00
Proposed method	2022	ImRMR with random forest classifier	Movement	**85.19**	**85.56**	**85.37**
Zhang et al. [42]	2018	Maximum joint mutual information algorithm with KNN classifier	Musk	79.52	22.89	51.21
Chen et al. [43]	2021	Self-learning feature selection with random forest classifier	Musk	88.63	53.73	71.18
Han et al. [40]	2021	Multi-objective particle swarm optimization with adaptive strategies with KNN classifier	Musk	83.80	–	–
Proposed method	2022	ImRMR with random forest classifier	Musk	**95.07**	**90.96**	**93.02**

Best performance in bold

### Discussion of using SFSFs for ranking feature subsets sequence

To further explore the effectiveness of the feature subset selected by the ImRMR, the sequence forward selection feature subset method (SFSFs) is used for the ranking feature subsets sequence to verify the pros and cons of the feature subset.

The steps of the SFSFs are as below.

1. The optimal feature subset starts with an empty set;
2. The first feature subset in the ordered feature subset set is added for the first time, and then the feature subset is added iteratively according to the order, which is combined with the selected feature subset to form a new feature subset;
3. This process continues until the classification accuracy of the newly mapped training set is greater than or equal to the raw classification accuracy, and the corresponding feature subset is the selected optimal feature subset.

**Fig. 5:**
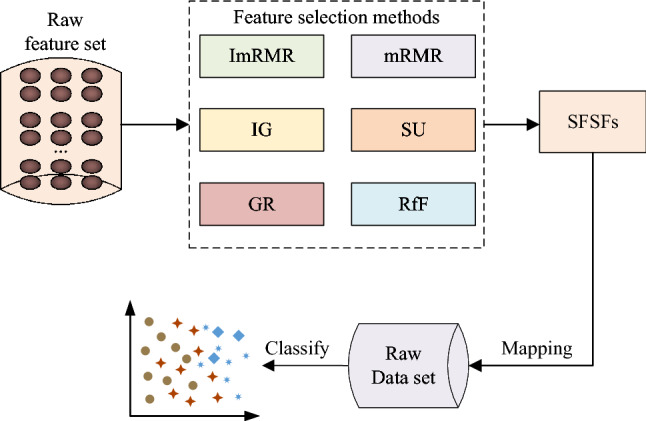
Feature selection method based on SFSFs

The feature selection method based on SFSFs is shown in Fig. 5. Combine the SFSFs search strategy to get the final preferred subset and mapping the optimal feature subset to the raw sample set can carry out classification and recognition. The effects of ImRMR and other five feature selection methods are compared by the dimensionality reduction rate, accuracy, comprehensive rate, recall, *F*-measure and precision when the classification accuracy with feature selection is greater than or equal to that of the raw datasets.

**Fig. 6:**
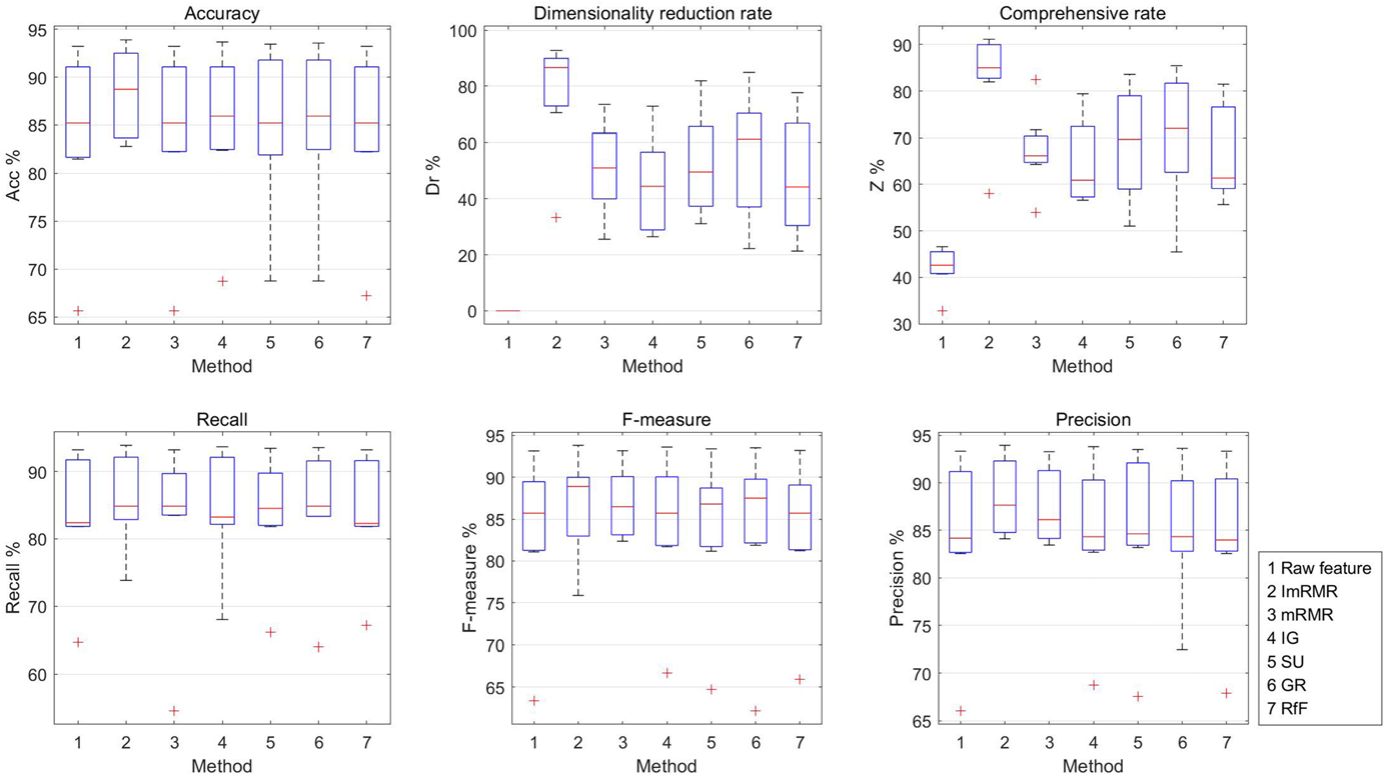
Comparison of the effects of six methods

The experimental results are shown in Table 5 and Fig. 6. And the abscissa labels 1, 2, 3, 4, 5, 6, and 7 in Fig. 6 represent raw feature and six feature selection method of ImRMR, mRMR, IG, SU, GR, and RfF, respectively.

**Table 5:** Experimental results of ImRMR and other five methods based SFSFs

Dataset	Performance (%)	Raw feature	Feature selection methods					
			ImRMR	mRMR	IG	SU	GR	RfF
Musk	Acc	85.21	**88.73**	85.21	85.92	85.21	85.92	85.21
	Dr	0.00	**92.77**	43.37	72.89	81.93	84.94	77.71
	Z	42.61	**90.75**	64.29	79.40	83.57	85.43	81.46
	Recall	**92.21**	**92.21**	89.61	**92.21**	89.61	90.91	**92.21**
	*F*-measure	87.12	**89.87**	86.79	87.65	86.79	87.50	87.12
	Precision	82.56	**87.65**	84.15	83.53	84.15	84.34	82.56
Urban	Acc	82.23	**84.77**	82.23	82.74	83.25	82.74	82.23
	Dr	0.00	**90.48**	51.02	54.42	51.02	61.22	44.22
	Z	41.12	**87.62**	66.63	68.58	67.13	71.98	63.23
	Recall	82.40	82.59	83.48	83.15	**84.52**	83.35	81.99
	*F*-measure	81.81	83.38	83.12	82.26	**83.39**	82.96	81.73
	Precision	84.18	**86.31**	84.75	84.33	84.63	84.20	83.98
Ionosphere	Acc	91.43	**93.33**	91.43	91.43	92.38	92.38	91.43
	Dr	0.00	70.59	**73.53**	26.47	70.59	70.59	70.59
	Z	45.71	81.96	**82.48**	58.95	81.48	81.48	81.01
	Recall	81.82	**84.85**	**84.85**	81.82	81.82	**84.85**	81.82
	*F*-measure	85.71	**88.89**	86.15	85.71	87.10	87.50	85.71
	Precision	90.00	**93.33**	87.50	90.00	93.10	90.32	90.00
Glass	Acc	65.63	**82.81**	65.63	68.75	68.75	68.75	67.19
	Dr	0.00	33.33	**66.67**	44.44	33.33	22.22	55.56
	Z	32.81	58.07	**66.15**	56.60	51.04	45.49	61.37
	Recall	64.62	**73.83**	54.49	68.02	66.12	63.96	67.14
	*F*-measure	63.36	**75.88**	61.47	66.69	64.75	62.13	65.95
	Precision	66.02	**84.26**	70.51	68.77	67.60	72.44	67.94
Movement	Acc	81.48	**83.33**	82.41	82.41	81.48	82.41	82.41
	Dr	0.00	**86.67**	25.56	31.11	31.11	44.44	34.44
	Z	40.74	**85.00**	53.98	56.76	56.30	63.43	58.43
	Recall	81.94	**83.72**	83.67	83.24	82.48	83.34	82.28
	*F*-measure	81.08	**82.83**	82.35	81.70	81.16	81.87	81.22
	Precision	83.03	**84.11**	83.45	82.70	83.19	82.33	83.57
PU	Acc	93.22	**93.89**	93.22	93.67	93.44	93.56	93.22
	Dr	0.00	**88.35**	38.83	28.16	49.51	69.90	29.13
	Z	46.61	**91.12**	66.03	60.91	71.48	81.73	61.17
	Recall	93.22	**93.89**	93.22	93.67	93.44	93.56	93.22
	*F*-measure	93.13	**93.80**	93.16	93.59	93.38	93.51	93.19
	Precision	93.33	**93.95**	93.28	93.81	93.51	93.63	93.33
Crane	Acc	**90.00**	**90.00**	**90.00**	**90.00**	**90.00**	**90.00**	**90.00**
	Dr	0.00	**80.00**	53.33	57.33	49.33	34.67	21.33
	Z	45.00	**85.00**	71.67	73.67	69.67	62.33	55.67
	Recall	90.32	**91.88**	89.73	91.80	89.81	91.80	89.81
	*F*-measure	90.24	90.02	90.08	**90.84**	89.24	90.51	89.71
	Precision	**91.59**	89.23	91.29	90.40	89.10	89.90	90.56

Best performance in bold

From Table 5 and Fig. 6, the performance of ImRMR based on SFSFs is better than five feature selection methods including mRMR, IG, SU, GR, and RfF in seven datasets. Among them, the comprehensive rate of ImRMR is 5.32–26.46%, 15.64–24.39%, 21.57–31.02%, 9.39–30.21%, 11.33–29.33% higher than the five feature selection methods on the five datasets of Musk, Urban, Movement, PU and Crane, respectively; the comprehensive rate of ImRMR on Ionosphere was only 0.52% lower than mRMR, and 0.48–23.01% higher than the other four methods; the comprehensive rate of ImRMR on Glass is 3.3% and 8.08% lower than that of RfF and mRMR, respectively, and 1.47–12.58% higher than the other three methods. The accuracy of ImRMR on six datasets including Musk, Urban, Ionosphere, Glass, Movement and PU is higher than the five feature selection methods, and the accuracy on Crane dataset is the same as the five feature selection methods. Furthermore, ImRMR outperforms the other five feature selection methods in recall, *F*-measure and precision on the seven datasets in most cases. Therefore, in a comprehensive comparison, with SFSFs the ImRMR feature selection method is superior to the five feature selection methods such as mRMR, IG, SU, GR, and RfF, and can effectively measure the contribution of feature subset to obtain an effective ranking feature subsets sequence.

## Limitations and future scope

This paper improves mRMR based on feature subsets, and proposes the ImRMR method, which is used to select efficient feature sets, reduce the dimension of feature sets, and improve the classification performance of samples. There still exists more issues to carry out in the future work, and some of the most important points are listed below:

- Extend the method to more datasets.
- This study currently proposes EGM to divide candidate feature subsets. Other more feature subset division methods are needed to be explored to improve the quality of candidate feature subsets.
- This study only uses Pearson correlation coefficient and mutual information to measure correlation and redundancy. In the next work, we will try to explore other measurement criteria to improve the effectiveness of the selected features.

## Conclusion

In this paper, considering the joint contribution between multiple features, the proposed ImRMR method expands the feature selection process based on feature subset.

EGM is used to divide the raw feature set into multiple different candidate feature subsets. Two criteria of Pearson’s correlation coefficient and mutual information are used to calculate the correlation and redundancy of candidate feature subsets, and weights are introduced to tradeoff both criteria. Then the SFS search strategy is combined for the ranking feature subsets sequence to obtain the optimal feature subset. Compared with five methods, including mRMR, InfoGain, Symmetrical Uncert, GainRatio, and ReliefF, experimental results verified the effectiveness on seven datasets. In most cases, ImRMR outperforms the other methods, and can effectively obtain the optimal feature subset and improve the performance of the classification.

## Data Availability

The datasets generated during and/or analysed during the current study are available from the corresponding author on reasonable request.
